# pH-dependent structural dynamics of neuropeptide Y in aqueous solution

**DOI:** 10.1371/journal.pone.0343614

**Published:** 2026-03-12

**Authors:** Hoa Thi Nguyen, Marc Spehr, Ana-Nicoleta Bondar, Paolo Carloni

**Affiliations:** 1 Forschungszentrum Jülich, Computational Biomedicine, INM-9, Wilhelm-Johnen Straße, Jülich, Germany; 2 Research Training Group 2416 MultiSenses – MultiScales, RWTH Aachen University, Aachen, Germany; 3 RWTH Aachen University, Physics Department, Aachen, Germany,; 4 RWTH Aachen University, Institute for Biology II, Department of Chemosensation, Aachen, Germany; 5 University of Bucharest, Faculty of Physics, Atomistilor, Magurele, Romania; University of Nova Gorica, SLOVENIA

## Abstract

Neuropeptide Y regulates key molecular processes in the brain. Its interaction with the cell membrane, where it binds to specialized receptors with key physiological roles, likely depends on pH. Available structural ensembles of both porcine and human peptides, solved by nuclear magnetic resonance (NMR) at an acidic pH in aqueous solution, indicate an *α*-helical core with unstructured termini. However, the protonation states of the carboxylic and histidine residues of the peptide, as well as the interplay between these states and peptide conformational dynamics, remain unexplored. In this study, we performed constant pH simulations and graph-based analyses to investigate the dynamics and H-bond patterns of neuropeptide Y within a pH range of 3.0 to 7.0. We found that an *α*-helical core is present at all pH values, though its length can vary by 2–3 residues depending on the pH. The pK_a_ of Asp16, part of the *α*-helix, and of Asp11 may shift by more than one pH unit. Based on these findings, we suggest that performing constant pH simulations may be required to accurately describe neuropeptide Y interactions with its cellular partners at the pH values of interest.

## Introduction

Neuropeptide Y (NPY) is a 36-residue peptide abundant in mammalian brains [1] (see Chart 1), where it is involved in regulating key processes such as memory formation and feeding [2]. In the hypothalamus, human (h)NPY participates in cell signaling pathways that delay aging [3]. In the peripheral nervous system, hNPY is involved in vasoconstriction [4]. The peptide can still be detected in *post mortem* human brain tissue [5], which allows studies of the hNPY distribution and concentration in neurodegenerative diseases. hNPY may bind to the cell membrane [6] and/or to its membrane-embedded target receptors [7,8]. These receptors are the Y1, Y2, Y4, and Y5 receptors, which belong to the G protein coupled-receptor (GPCR) superfamily [9–11]. hNPY-based peptide conjugates (i) are being designed for cancer diagnosis because the receptors targeted by hNPY are overexpressed in cancer cells [12], which often display dysregulated pH [13]. These conjugates are also used to deliver therapeutic cargo to these cells [14–16]. pH likely affects NPY’s membrane interactions substantially, as suggested by two observations: **(i)** Electrostatic interactions, which drive peptide binding to the membrane [17], are weaker in a buffer at pH 7.4 than in aqueous solution at pH 7.0 [18]. **(ii)** The estimated free energy of peptide binding to a standard membrane is approximately 40% of the energy calculated for a corresponding membrane with a high concentration of acidic lipids [19]. These observations raise the key question whether and, if so, how conformational dynamics of NPY depend on pH, which in turn could influence its membrane and receptor binding. To study conformational peptide dynamics as a function of pH, here we carry out atomic-level simulations using constant pH and standard molecular dynamics simulations. We rely on graph-based approaches to characterize the protonation-dependent dynamics of the peptide.

The three-dimensional folds of NPY and of the two other peptides from the pancreatic polypeptide family, the pancreatic peptide (PP) and peptide YY (PYY), have been studied for decades [20]. The first structure of the PP peptide from turkey (avian, aPP), solved in the early 80’s by X-ray crystallography, revealed the PP fold in this family [21]. It consists of the following regions: (i) an N-terminal region with a polyproline type-II helix (residues 2–8) containing the three conserved Pro residues (see P2, P5, and P8 in Chart I); (ii) an amphipathic α-helical core (residues 14–31); and (iii) a flexible C-terminal segment [21–23]. The N-terminal segment is back-folded anti-parallel to the *α*-helical segment, such that the peptide assumes a hairpin structure. The three conserved N-terminal Pro residues are on the same side of the polyproline helix and interdigitate with hydrophobic residues of the α-helical core helix, a structural feature suggested to explain why a monomeric PP peptide would be stable in solution. Intramolecular H-bonds involving charged residues were suggested to help shield the hydrophobic core from the aqueous solution [24].

**Fig 1:**
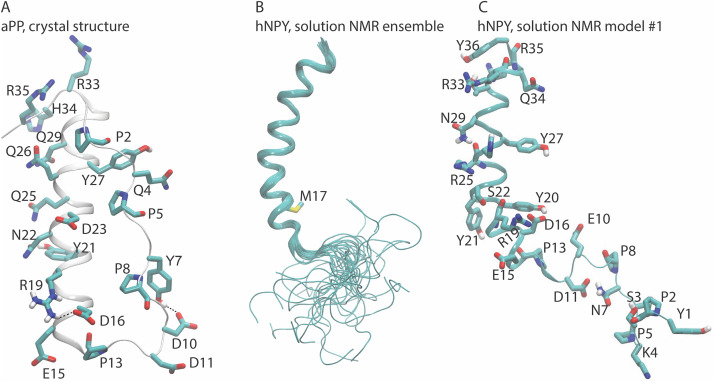
Architecture and intramolecular H-bonding of aPP and NPY peptides. (A) The aPP X-ray structure, solved at high resolution, reveals intramolecular H-bonds between Y7 and D10 and between D16 and R19 (PDB ID 2BF9). This panel was created using Visual Molecular Dynamics (VMD) [34]. For clarity, only selected H atoms and the backbone of the proline residues are shown. (B) The NMR structural ensemble (26 conformations) of hNPY in aqueous solution, taken from PDB ID 1RON. The N-terminal region is flexible and lacks the tight packing against the α-helical core observed in the aPP crystal structure from panel A. M17 distinguishes hNPY from pNPY (Chart 1), which was investigated in this work. (C) Model #1 from the NMR ensemble with selected residues. In this model, the distances are as follows: S22-Oγ and the R19 backbone carbonyl group: 2.8 Å; N29-Nδ2 and R25 (O): 3.2 Å; S3-Oγ and P5 (O): 4.6 Å; the side chains of N7 (Nδ2) and D11(Oδ1): 2.8 Å; D16 (Oδ2) and Y20 (O): 2.8 Å; and T32 (Oγ) and Y36 (OH): 3.4 Å, D6 (Oδ1) and P5(O): 4.8 Å.

Fig 1A illustrates the intramolecular hydrogen (H) bonds revealed by the X-ray crystal structure of aPP, which was solved at a very high resolution of 0.99 Å [22]. Tyr7 and Asp10, and Asp16 and Arg19, are within H-bonding distance of 2.7–2.8 Å. In a more recently solved X-ray crystal structure of an antibody bound human (h)PYY peptide, the amidated C-terminal region of the peptide H-bonds to the Fab antibody. It is thought that such H-bonds might be more generally relevant for the binding of PP peptides to their membrane receptors [20]. Tyr20 and Arg25 of the *α*-helical core are important for the conformational dynamics of NPY: when either or both residues are mutated to Phe in hNPY, the peptide exhibits higher thermal stability and a higher propensity to oligomerize in aqueous solution [25].

The NMR structure of hNPY in solution at pH 3.2 and 310 K indicates an α-helical core from Pro13 to Thr32 [26] (Fig 1B), and a highly flexible N-terminal region. There is no close packing between the latter and the α-helical core, unlike the crystal structure of pYY (Fig 1A and 1B). As was initially observed for pYY, the NMR structure of hNPY reveals intramolecular H-bond distances between side chains – specifically, between Asp16 and Tyr20, as well as between Arg35 and Tyr36 (Fig 1C). Additionally, the ensemble of NMR structures of hNPY also indicates that intramolecular H-bonding between carboxylic side chains and backbone carbonyl groups can be sampled at acidic pH. In one of the structural models of the ensemble (#26), the carboxyl group of Asp6 is within 3.0–3.1 Å of the backbone carbonyl groups of Lys4 and Pro5, and the carboxyl group of Asp11 is within 2.8 Å distance of the backbone carbonyl group of Pro8. Such short distances between a carboxylic group and a backbone carbonyl group could suggest that the former is protonated.

Given the important role of pH in NPY’s interactions with cellular partners and membranes, we studied here the conformational dynamics of the peptide as a function of the pH using constant pH molecular dynamics simulations, CpHMD [27,28]. CpHMD simulations allow the protonation states of ionizable groups to respond to changes in the chemical environment and external pH. This contrasts with standard MD, which uses fixed protonated states for ionizable side chains and it may provide limited information on peptide conformational dynamics when pK_a_ values of residues are near the chosen pH (as is the case for histidine at physiological pH). For these residues, both protonated and deprotonated states can coexist, and their relative amounts can change due to conformational rearrangement. We use graph-based approaches [29,30] to characterize the protonation-dependent dynamics of the peptide.

In light of our long-term goal of studying peptide/membrane binding, we chose porcine (p)NPY as our model system. This decision was made for the following reasons: (i) an available solution NMR structure of pNPY at pH 3.1 revealed very similar features [31]; (ii) hNPY and pNPY differ only by the M17L substitution [26] (see Chart 1 and Fig 1B) and (iii) the structure of pNPY bound to micelles has been determined [32].

**Chart 1.** The hNPY and pNPY sequences. We consider residues 29–64 (without the signal peptide and C-terminal residues 65–97 of the full length NPY, UniProt entry A4D158), labeled 1 starting from the N-terminal Tyr, such that Y1 corresponds to Y29 in full length sequence of NPY [33]. The C-terminus is amidated [26]. Note that the sequence of hNPY and pNPY are identical except at position 17 (Met in hNPY and Leu in pNPY). Asp and Glu residues, Arg and Lys, the histidine are colored red, blue, and green, respectively. The underlined residues belong to the helical segment of the peptide.


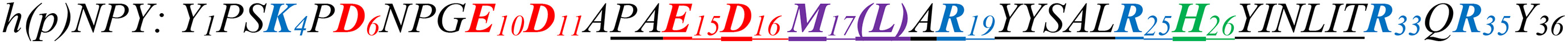


## Methods

Our calculations are based on the hNPY NMR structural ensemble (model #1) in solution (PDB ID 1RON) [26]. The pNPY structure was obtained by replacing Met17 with a Leu residue. The Phyre2 web portal was used to model the mutant peptide [35]. During the equilibration phase of standard MD simulations, His26 was protonated in Nδ tautomer and Glu and Asp residues (Chart 1) were considered negatively charged. For the constant pH simulations, the protonation of these residues is dictated by the algorithm. The peptide was placed in the center of coordinates of a box of 14,125 water molecules of 77 x 77 x 77 Å^3^ with 0.15 M neutralizing NaCl using the CHARMM-GUI protocol [36].

*Force-fields.* For the standard MD simulations*,* we used the CHARMM36m force field parameters for the peptide and ions [37–42], and the TIP3P water model [43]. This combination of force fields is recommended for this approach [37,44,45]. For the CpHMD simulations we used the recommended protocol [46] whereby the protein is described with the CHARMM22/CMAP force field [40,47], water molecules, with the CHARMM-modified TIP3P model [48], and ions, with CHARMM36 [42]. Thus, a different water model was used in the two types of simulations in order to comply with the recommended protocols.

*Standard MD simulation.* We used the SHAKE algorithm [49] to constrain all covalent bonds involving H atoms, and an integration timestep of 2 fs. Following geometry optimization of the peptide using conjugate gradient [50], we performed a 10 ns equilibration in the *NVT* ensemble (constant number of atoms *N*, constant volume *V*, and constant temperature *T*) with harmonic constraints of 1.0 kcal/mol/Å^2^ on the peptide hetero-atoms. We used for this phase the Langevin thermostat [51] to keep constant temperature conditions. The damping coefficient was 5.0 ps^-1^, and the target temperature was set at 303.15 K. Hydrogen atoms were kept uncoupled from the thermostat to avoid noise. This reduces artifacts because of their fast movements [52]. We then switched off all constrains and performed simulations at a constant temperature of 303.15 K, with the same setup as above, and at a constant pressure of 1 atm using the Langevin piston (Nose-Hoover Langevin barostat) [53]. The target pressure was set at 1.01325 bar (1 atm), with an oscillation period of 50 fs. Long-range electrostatics interactions were calculated using the Particle Mesh Ewald (PME) method within 1Å of grid spacing [54] and a spline order of 6. The cutoff the radius of van der Waals interaction and the real part of the electrostatics was set to 12 Å. We used the same starting coordinates to perform three *NPT* production runs, 500 ns-long each, with NAMD [55] package. The simulations differ in their Maxwell-Boltzmann velocity distributions.

*CpHMD simulations.* The initial structure was a snapshot after 465 ns of the standard MD simulation above. During the dynamics, the charge of a titratable residue may change. Because the Ewald summation requires the simulation box to remain neutral [56], we used the protocol of Wallace et al., [57], whereby the protonation titration is coupled with the simultaneous ionization or neutralization of a dummy co-ion in solution.

The CpHMD simulations utilised the Nose-Hoover thermostat [58] and barostat [59] to maintain constant temperature and pressure, respectively. In the thermostat, the target temperature was set at 303.15K, and the mass of the thermostat was set at 1000 kcal.ps^2^. In the barostat, the reference pressure was set at 1 atm, the mass of piston of 500 amu. The friction coefficient for the piston was set to 20 ps^-1^. The temperature of the bath was maintained at 303.15 K. The SHAKE algorithm [49] was used to constrain all covalent bonds involving hydrogen (H) atoms. We then switched off all constrains and performed simulations at a constant temperature of 303.15 K and pressure of 1atm, with the same setup. Following the recommended minimization procedure for CpHMD simulations in CHARMM [46], we performed geometry optimization of the peptide within 10,000 steps of Steepest Descents (SD) and 5,000 steps of an adopted Newton-Raphson method (ABNR). Likewise, following the recommended protocol, we equilibrated pNPY in water using 3 stages as follows: **(i)** heating by 0.2 ns restrained MD from 5 K to 303.15 K, in 100,000 steps with harmonic force constant of 1.0 kcal/mol/Å^2^. **(ii)** Four CpHMD equilibration steps of 0.2 ns each: the first two in the *NVT* ensemble, and the last two in the *NPT* ensemble, with the peptide hetero-atoms restrained by a harmonic force constant of 1.0, 0.5, 0.1, and 0 kcal/mol/Å^2^. **(iii)** CpHMD productions were performed without any constraints.

For each pH value, we used the equilibrated structure to perform three independent CpHMD simulations. We name these simulations as replica R#1 (50 ns), R#2 (50 ns), and R#3(40 ns).

*Predictions of pKa’s values of titratable residues.* The titration coordinate λ takes values λ = 1 for the deprotonated state, and λ = 0 for the protonated state [27]. Since histidine and carboxyl sidechains each contain two titration sites (denoted as tautomeric states), an additional coordinate, *χ*, is used to describe the interconversion between the tautomeric states. The numbers of deprotonated ($N_{deprot,\;i}$) and protonated states ($N_{prot,\;i}$) for a residue *i* are then defined as:


$$N_{deprot,\;i}=N\left(\lambda_{i}\geq \text{0}.\text{8};\chi_{i}<\text{0}.\text{2}\;or\;\chi_{i}>\text{0}.\text{8}\right)$$ (1)

$$N_{prot,\;i}=N\left(\lambda_{i}\leq \text{0}.\text{2};\chi_{i}<\text{0}.\text{2}\;or\;\chi_{i}>\text{0}.\text{8}\right)$$

Mixed tautomeric states with $\text{0}.\text{2}<\chi_{i}<\text{0}.\text{8}$ are discarded. We use cut-off values $\lambda_{i}\epsilon [\text{0}.\text{8},\text{1}.\text{0}]$ for deprotonated states, and $\lambda_{i}\epsilon [\text{0}.\text{0},\text{0}.\text{2}]$ for protonated states. The charge of atom *α* on a titrating residue *i* reads:


$$q_{i,\alpha }=\lambda_{i}q_{i,\alpha }^{d}+(\text{1}-\lambda_{i})q_{i,\alpha }^{p}$$ (2)

where $q_{i,\alpha }^{d}$ and $q_{i,\alpha }^{p}$ are the charges in the deprotonated and protonated states, respectively.

For a trajectory at a specific pH, and for a residue *i* (in this study, *i* = 1 for Asp6, *i* = 2 for Glu10, *i* = 3 for Asp11, *i* = 4 for Glu15, *i* = 5 for Asp16, and *i* = 6 for His26), the number $N_{prot,\;i}$ counts the number of times the titratable residue *i* in the protonated state ($\lambda_{i}\leq \text{0}.\text{2}$), while $N_{deprot,\;i}$ counts the number of times the titratable residue *i* in the deprotonated state ($\lambda_{i}\geq \text{0}.\text{8}$). The deprotonated fraction of residue $S_{i}$ can be calculated from the simulations at different pH as:


$$S_{i}=\frac{N_{deprot,i}}{\left(N_{deprot,i}+N_{prot,i}\right)}$$ (3)

where $N_{deprot,i}$ and $N_{prot,i}$ are number of deprotonated and protonated states, respectively. The *titration curve* plots $S_{i}(pH)$ as a function of the pH, is obtained by fitting the $S_{i}$ values across all simulated pH values to the generalized Henderson-Hasselbalch (HH) equation,


$$S_{i}(pH)=\frac{\text{1}}{\text{1}+{\text{1}\text{0}}^{n\left({pK}_{a,i}-pH\right)}}$$ (4)

where *n* is the Hill coefficient.

The ${pK}_{a,i}$ value of residue *i* is given by the pH value at which $S_{i}$ = 0.5.

*H-bond network analysis.* To study how the internal H-bond network of the peptide as a function of the pH, we used a graph-based algorithm and a graphical user interface, Bridge/Bridge2 [29,30]. With this approach, an H-bond graph consists of nodes, i.e., the H-bonding protein groups included in the graph computation, and edges, i.e., the H-bond connections (direct or water mediated) between these NPY groups. We used the last 2,000 equally spaced coordinate snapshots from 40 ns of each of the CpHMD labeled as R#1 and R#2, as well as the last 1,500 equally snapshots from 30 ns of the CpHMD labeled as R#3. Bridge computations were performed on 3 replicas simultaneously by uploading all trajectories for a given pH. To understand how side chains, backbone groups, and water-mediated bridges, contribute to the intramolecular H-bond network of pNPY, we separately computed three sets of H-bond graphs:

1) Direct H-bonds of side chains and backbone groups, and water-mediated bridges of these groups.
2) Direct side chain to sidechain H-bonds and water-mediated bridges between side chains.
3) Direct H-bonds between side chains.

We considered water bridges between an H-bond donor and an H-bond acceptor formed by one, two or three H-bonded waters. To identify H-bonds we used standard geometric criteria: the distance between the donor and acceptor heteroatoms ≤ 3.5 Å; the H-bond angle lower than either 20º or 60º. Results for both cutoff values are reported. The *occupancy* of an H-bond is given by the percentage of coordinate sets, out of the total number of coordinate sets used for analyses, in which the H-bond criteria are met. Here, we consider only H-bonds with an occupancy equal to or higher than the minimum thresholds reported in Table 1.

**Table 1:** H-bond criteria and occupancy thresholds used for the H-bond graphs.

Set of H-bond graphs	H-bond angle criterion of 20^0^	H-bond angle criterion of 60^0^
1)	10%	50%
2)	10%	25%
3)	10%	15%

Notice that Bridge does not distinguish between the H-bonds that a protein group donates or accepts.

*Secondary structure elements.* These were determined using STRIDE (TIMELINE analysis) in the Visual Molecular Dynamics (VMD) [34] graphics software.

## Results

### pKa computations

S1–36 Figs in S1 File show the evolution of coordinates, partial charges and cumulative deprotonated fraction values of each titratable residues *i* of the peptide (*i* = 1 for Asp6, *i* = 2 for Glu10, *i* = 3 for Asp11, *i* = 4 for Glu15, *i* = 5 for Asp16, and *i* = 6 for His26), informing the convergence of protonated states. For **Asp6** (S1–S6 Figs in S1 File), at pH = 7, 6 $\lambda_{\text{1}}$values across the three simulation replicas are close to 0.8–1.0 (S1 and S2 Figs in S1 File). The deprotonated fraction of residue $S_{\text{1}}$ is close to 1 (S6 Fig in S1 File), hence this residue is fully deprotonated. At pH = 5, 4, $\lambda_{\text{1}}$values are found both in the range of 0.8–1.0 (more at pH 5 than pH 4), and in the range of 0.0–0.2 (more at pH 4 than pH 5). As a result, Asp6 is mostly deprotonated at pH = 5 with $S_{\text{1}}\sim \text{0}.\text{8}$ (S3 and S6 Figs in S1 File) and it is mostly protonated at pH = 4 with $\text{0}.\text{2}\leq S_{\text{1}}\leq \text{0}.\text{4}$ (S4 and S6 Figs in S1 File). At pH = 3, $\lambda_{\text{1}}$ values are primarily distributed in the range of 0–0.2 and $S_{\text{1}}\sim \text{0}.\text{1}$, indicating that Asp6 is fully protonated (S5 and S6 Figs in S1 File).

**Glu10** is deprotonated at pH values of 7, 6, and 5 ($S_{\text{2}}\sim \text{1}.\text{0}$, S7–S9 Figs in S1 File). At pH = 4 and 3, it is mostly deprotonated or protonated ($S_{\text{2}}\sim \text{0}.\text{6}-\text{0}.\text{8}$, and $S_{\text{2}}\sim \text{0}.\text{2}-\text{0}.\text{4}$, respectively, S10–S12 Figs in S1 File). **Asp11** is deprotonated at pH = 7 and 6 ($S_{\text{3}}\sim \text{1}.\text{0}$ and $S_{\text{3}}\sim \text{0}.\text{8}$, respectively, S13, S14 and S18 Figs in S1 File). At pH = 5, it is mostly protonated ($S_{\text{3}}\sim \text{0}.\text{4}$, S15 and S18 Fig in S1 File). At pH = 4 and 3, it is protonated ($S_{\text{3}}\sim \text{0}.\text{1}$ and $S_{\text{3}}\sim \text{0}.\text{0}$, respectively, S16–S18 Figs in S1 File).

**Glu15** is fully deprotonated at pH = 7, 6, 5 ($S_{\text{4}}\sim \text{1}.\text{0}$, S19–S21 and S24 Figs in S1 File) while at pH = 4 it is mostly deprotonated ($S_{\text{4}}\sim \text{0}.\text{6}$). It is protonated at pH = 3 ($S_{\text{4}}\sim \text{0}.\text{2}$, S22–S24 Figs in S1 File). **Asp16** is deprotonated at pH = 7–4 ($S_{\text{5}}\sim \text{0}.\text{8}-\text{1}.\text{0}$, S25–S28 and S30 Figs in S1 File), and it can be either deprotonated or protonated at pH = 3 ($S_{\text{5}}\sim \text{0}.\text{4}-\text{0}.\text{6}$, S29–S30 Figs in S1 File). **His26** is mostly neutral at pH 7 ($S_{\text{6}}\sim \text{0}.\text{8}$, S31 and S36 Figs in S1 File). At pH = 6, it is both mostly positively charged and neutral ($S_{\text{6}}\sim \text{0}.\text{4}$, S32 and S36 Figs in S1 File). At lower it is totally protonated ($S_{\text{6}}\sim \text{0}.\text{0}-\text{0}.\text{1}$) (S33–36 Figs in S1 File).

**Fig 2:**
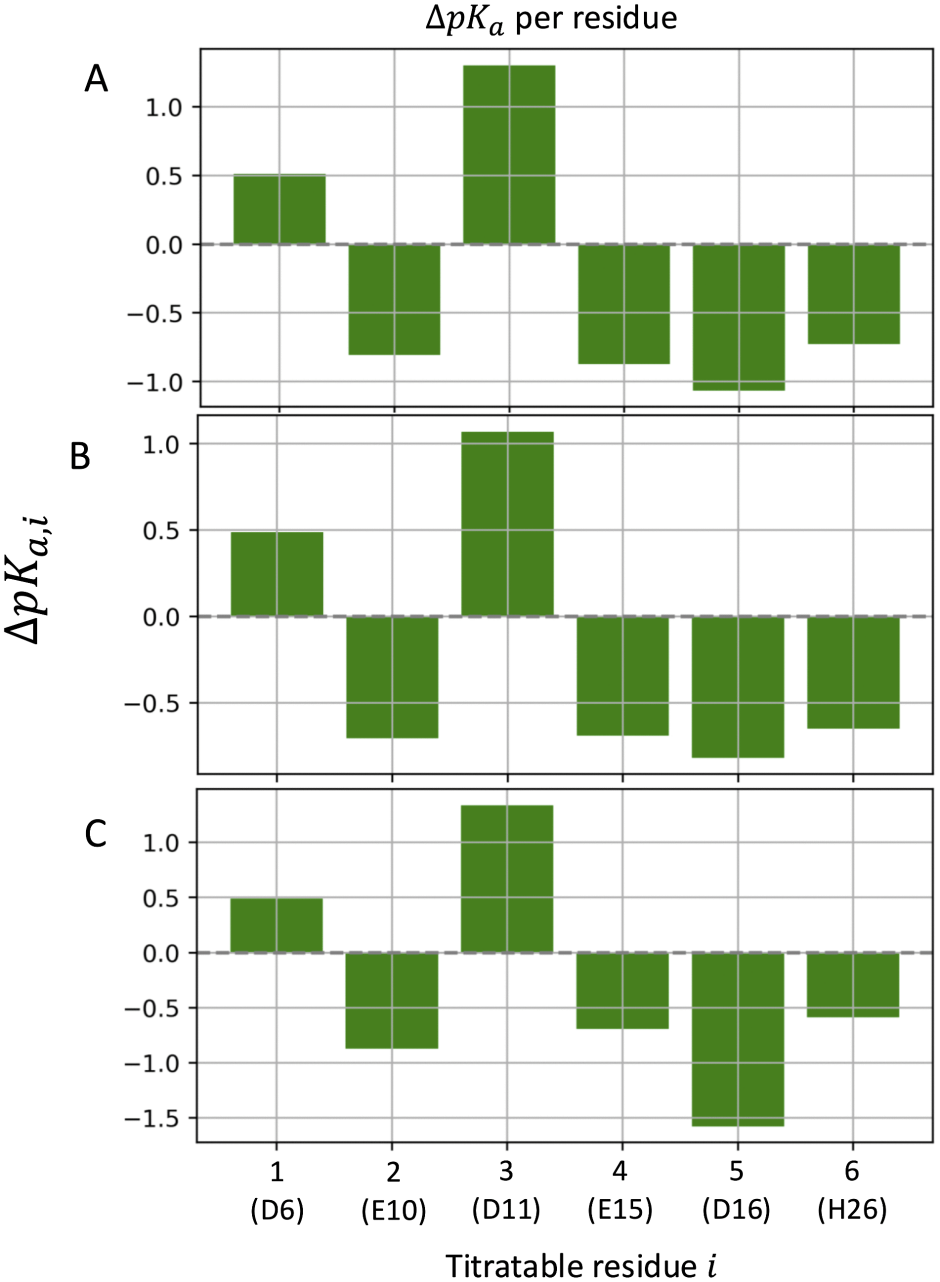
$\Delta {pK}_{a,i}$ values of the titratable residues (i: 1, ..., 6). (A-C) refer to simulations R#1–3.

**Fig 3:**
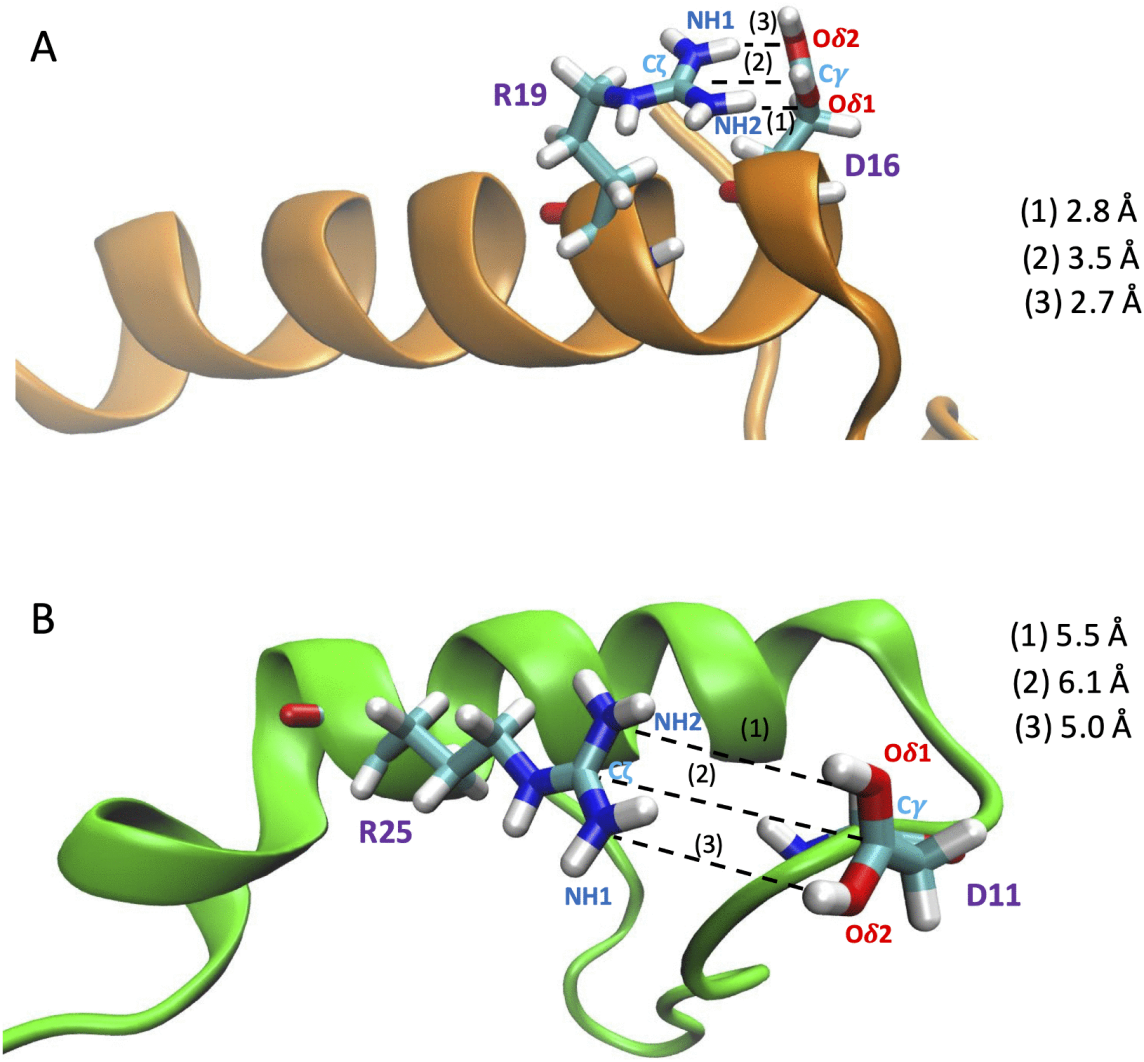
Asp16 - Arg19 (A) and Asp11 – Arg25 (B) salt bridge from our CpHMD simulations at pH 7 (R#1, 2, respectively). In these snapshots (at 91.8 ns and 135.0 ns, respectively), the (1) Oδ1 (Asp) – NH2(Arg), (2) Cγ(Asp) – Cζ(Arg), and (3) Oδ2(Asp) – NH1(Arg) distances are the shortest. The first salt bridge is also observed in R#2 and R#3, whereas the second only in R#2 at pH 7, 6.

The pK_a_ values, estimated from the titration curves (S37–S38 Figs in S1 File, S1 Table), turned out to be converged within 10 ns of constant pH dynamics. The largest shifts in pK_a_ (ΔpK_a,i_) of residues *i* (X_i_) relative to its “standard” value in the CH3CO-AAXAA-NH2 [46] peptide are observed for Asp16 (ΔpK_a,5_ < −1) and Asp11 (ΔpK_a,3_ > 1) (see the Fig 2 and S1 Table). The negative pK_a_ shift of Asp16 might be caused, at least in part, by its salt bridge with Arg19 (Fig 3A and S39–S63 Figs in S2 File). At pH = 5 or lower, Asp11 interacts with Glu10, that is mostly deprotonated, as seen in the above analysis. The ionized state of the residue may be stabilized by its salt bridge with Arg25 (Fig 3B and S55 Fig in S2 File). The Asp11-Glu10 interaction may cause, at least in part, by the large positive shift in pK_a_.

### H-bond networks

We used the Bridge/Bridge2 graph-based algorithm and graphical user interface [29,30] to identify H-bond networks at different pH investigated here (Figs 4–7, S39–S63 in S2 File, S2–S4 Tables). We performed separate computations for H-bond networks for protein groups (side chains and backbone), focusing on either direct or water mediated H-bonds (up to three water molecules, Figs 4–5), side-chain to side-chain interaction involving either direct or water mediated H-bonds (Fig 6), and side-chain to side-chain interaction involving direct H-bonds (Fig 7). All graph computations were performed with a distance H-bond criterion of 3.5 Å. To test how the choice of the H-bond angle (between acceptor, hydrogen atom and donor) influences the results used two different angle criteria, 20^0^ and 60^0^. As expected, using the stricter 20^0^ H-bond angle criterion reduced the number of H-bonds in the graphs (Figs 5, 6B, 7B and S1 Text).

**Fig 4:**
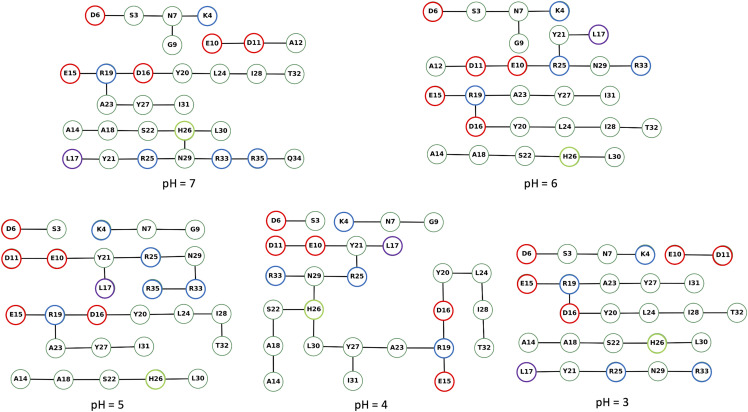
H-bond graphs computed for backbone, side chains, and three water bridges at different pH with an H-bond angle criterion of 60°. We used the complete dataset of three replicas at each simulated pH value to compute the H-bond graphs with Brigde2 [29]. All graph computations used an H-bond distance criterion of 3.5 Å. The color coding of the nodes is as follows: red for Asp and Glu residues, blue for Arg and Lys, violet for Leu17, lime for His26, and green for all other residues. The H-bond occupancy threshold was set at 50%. Note that nodes and edges of the graph were manually arranged in the Bridge2 graphical interface to minimize the space taken by each panel; thus, nodes representing the residues of the peptide are not arranged according to their relative location in the peptide.

**Fig 5:**
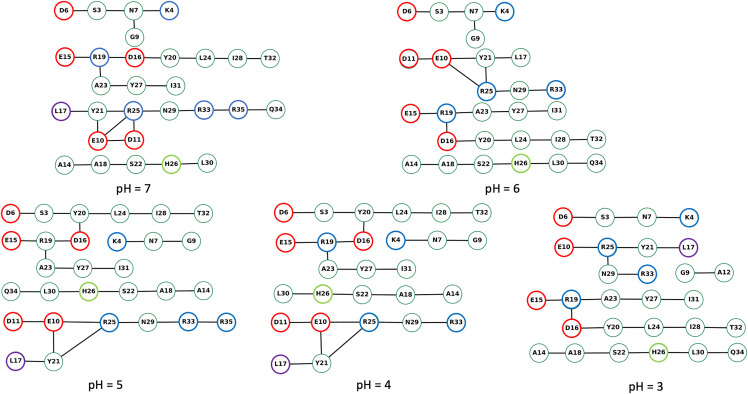
Same as Fig 4, here with an H-bond angle criterion of 20°. The H-bond occupancy threshold was set at 10%.

**Fig 6:**
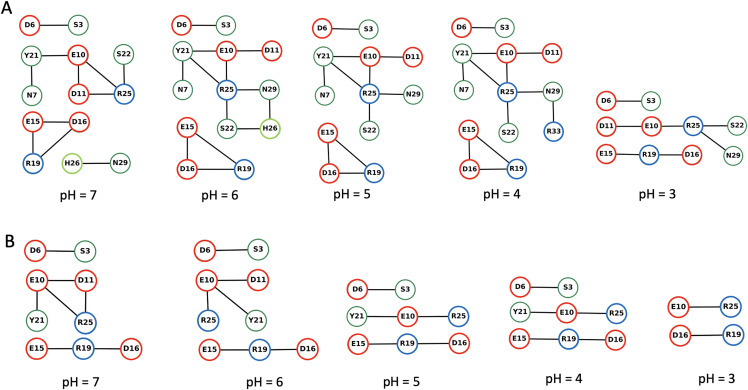
H-bond graphs for direct side-chain to side-chain H-bonds and water-mediated bridges, without the protein backbone groups. We used the complete dataset of all replica simulations at each pH value. (A, B) H-bond graphs with an H-bond angle criterion of 60° in (A) and 20° (B). The H-bond occupancy threshold used, 25% and 10% for the angle criterion of 60° and 20°, respectively.

**Fig 7:**
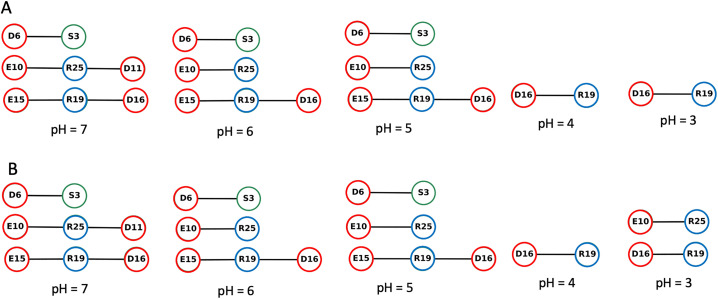
Same as Fig 4, but only for direct H-bonds of sidechains. The H-bond occupancy threshold used, 15% and 10% for the angle criterion of 60° and 20°, respectively.

The number of direct H-bonds (Fig 7A) is lower than that of water-mediated H-bonds, whether one considers only side chains (Fig 6A) or side chains and protein backbone groups (Fig 4). At all pH values and with either H-bond angle criterion, Asp16 side chain H-bonds to Arg19 side chain (Fig 7A), while the side chains of Asp6, of Glu10, and of Glu15 form direct H-bonds with Ser3, Arg25, Arg19 at pH 7−5 (Fig 7A), but not at pH < 5 (Fig 7A).

H-bonds and water-mediated bridges between side chains are as follows. **Asp6** samples water- mediated H-bonds with Ser3 at all pH values, and direct H-bonds at pH 7−5 (Figs 6A, 7A). At all pH values, **Glu10** has direct or water mediated H-bonds with Arg25 and water-mediated H-bonds with Asp11 (Fig 6A). **Asp11** has direct or water mediated H-bonds with Arg25 at pH 7 (Fig 6A). **Glu15** has direct or water-mediated H-bonds with Arg19 at all pH values (Fig 6A), and water- mediated H-bonds with Asp16 at pH 7−4 (Fig 6A). **Asp16** connects to Arg19 side chain at all pH values (Fig 6A).

### Conformational dynamics

Our starting model of pNPY in solution is based on the hNPY NMR structure at a pH of 3.2 and a temperature of 310 K(26). Its *α*-helix extends from residue Asp11 to Tyr36 [26]. Such an α-helical segment is mostly maintained during standard MD simulations (500-ns long MD simulations at 303.15 K, see S64 Fig in S3 File, S5 Table) and during the constant pH simulations at 303.15K (S65–S69 Figs in S3 File). However, residues Arg33-Tyr36 at the C-term and Asp11-Ala14 at the N-term lose their secondary structure (S65–S69 Figs in S3 File). Thus, the helix becomes shorter. A decrease of *α*-helical content relative to the human one has been observed also in NMR studies of pNPY in solution [31]: at pH 3.2 and a temperature of 310 K, the helix extends from Pro13 to Tyr36.

**Fig 8:**
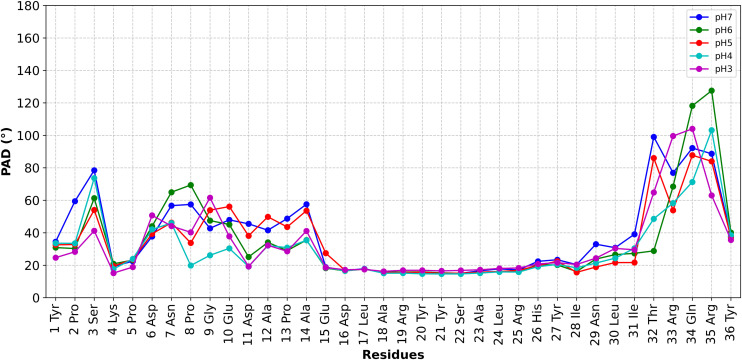
Backbone fluctuations during the CpHMD simulations as quantified by PAD values. The larger the PAD values, the larger the fluctuations of the residue backbone atoms [60]. The central helical core has very similarly low PAD values, indicating similar rigidity, whereas the N- and C-termini have larger fluctuations with distinct PAD values in each of the three replicas.

The conformational fluctuations of the peptide are quantified by PAD [60] values (Fig 8). The higher the PAD value for a backbone unit, the larger its fluctuation. Overall, as expected, residues at the N- and C-termini (mostly coils) have larger fluctuations than the helix at all values of pH, and even more so at higher and low pH values (S70–S73 Figs in S3 File). An unanticipated finding is that only a smaller fragment of the peptide, from Glu15 to Arg25, has very small PAD values that are similar to those of an α-helical segment of a folded protein, as seen in refs [61–65].

## Discussions and conclusions

We have carried out constant pH simulations to study the protonation patterns of pNPY in aqueous solution, upon acidification, from pH 7 to 3. Three independent simulations, for a total of 110 ns, indicate that the peptide keeps most but not all of its long *α*-helical structure present at pH 3.1 and pH 7.4 for the same peptide, as observed by NMR [31], while few residues at both the N-terminus and the C-terminus side assume random coil conformations (S65–S69 Figs in S3 File). As expected, the helix is relatively rigid at all pH values (Fig 8). The termini are disordered, as observed experimentally in both the human [26] and porcine [31] NMR structural ensembles.

Our H-bond graph computations indicate that the intra-molecular H-bond network of the peptide is strongly pH dependent. As anticipated from the NMR structure, there are relatively few H-bonds directly between the side chains, and Asp16 and Arg19 directly connect to each other at each pH value (Fig 7). Asp6 and Glu15 lack direct sidechain H-bonds at pH = 3 and pH = 4. H-bonds between Asp carboxyl groups and Pro backbone carbonyl groups are instead present only in the hNPY NMR ensemble.

The pKa values of the six ionizable residues of the peptide estimated from their calculated titration curves in aqueous solution (S37–S38 Figs in S1 File) converged within about 10 ns. Two residues experience rather large changes in pKa (more than 1 pKa unit) relative to their standard values, as assumed to be in the CH3CO-AAXAA-NH2 [46] peptide, where X is the residue we focus on [46]. Asp16 has a low pKa value, possibly because of its close interactions with Arg19 (Fig 3A). As a result, Asp 16 is always deprotonated except at pH = 3. The opposite effect is observed for Asp11, which is adjacent to Glu10, and which Asp 11 is protonated even at mildly acidic solutions. The only histidine residue of the peptide, His26, is mostly neutral only at pH 7, and it is protonated at lower pH values.

In the NMR ensemble of conformations of hNPY, model #1 features one intermolecular H-bonds that is also present in our simulation. This involves Glu10 (N) and Asp11 (Oδ1)). However, several other interactions are present only in the simulated structural ensemble (including Asp16 (Oδ1) with Arg19 (NH1), Asp6 (N) with Ser3 (Oγ), Asp6 (N) with Ser3 (Oγ), Glu15 (Oε1) with Arg19 (N)) and only in the NMR structure (Asp11 (Oδ1) with Asn7(Nδ2), Asp16(Oδ2) with Tyr20(OH)).

In conclusion, we have presented here a CpHMD study of NPY in neutral and acidic solutions, ranging from pH 7.0 to pH 3.0. The pK_a_ values of Asp residues and the protonation states of ionizable residues vary significantly upon decrease of pH. We suggest that performing CpHMD simulations may be critical for accurately describing NPY binding with its cellular partners, including the Y1R/Y2R receptors and cellular membranes, at physiological and acidic pH. Indeed, constant-pH molecular dynamics captures the coupling between protonation equilibria and the conformational dynamics that govern peptide binding, interaction specificity, and folding. They make take into account pH-dependent electrostatics and binding-induced pKa shifts which are very difficult to access with fixed-protonation simulations. CpHMD can predict pH-dependent salt bridges and charge patterning that control the folding of short peptides, such as those in ref [66]. This approach could also be useful in peptide design studies, such as that in ref [67]. Here, CpHMD simulations could identify binding-induced pK_a_ shifts and protonation networks that stabilize peptide–target complexes. This might enable the rational tuning of affinity and multi-target selectivity through peptide design. Finally, CpHMD simulations could help characterize putative interaction regions in the target protein, as in the study of ref [68]. Indeed, CpHMD can reveal pH-dependent interaction hotspots by tracking changes in surface residue protonation upon mutual interaction, thereby improving simple descriptions based on static structural contacts.

## Supporting information

S1 File Prediction of the pKa values of titratable residues. Here, we present our calculations of the protonation coordinate *i*, partial charge, and deprotonated fraction S_*i*_ of residue *i*, *i* = 1 for Asp6, *i* = 2 for Glu10, *i* = 3 for Asp11, *i* = 4 for Glu15, *i* = 5 for Asp16, and *i* = 6 for His26.
(ZIP)

S2 File H-bond networks. (ZIP)

S3 File NPY conformation dynamics. Standard MD simulations. Here all the titratable side chains were in their standard protonation states, as chosen at the beginning of the simulation. During the final 110 ns of each of the three MD simulations (lasting 500 ns), the α-helical segment (Glu15–Ile31), was conserved whereas the N- and C-terminal regions were rather disordered. A summary of the three analyses is offered in S5 Table and S64–S69 Figs in S3 File.
(ZIP)

S1 Table Mean pKa values obtained from our simulations. (PDF)

S2 Table Occupancies of either direct H-bonds or water-mediated bridges among entire residues (that is, backbone and side chains), averaged over the constant pH simulations at different pH values. This and the next two tables have as occupancies as those in S39–S49 Figs in S2 File (50% and 10% for criteria of 60° and 20°, respectively), S50–S56 in S2 File (25% and 10%, S3 Table), and S57–S63 in S2 File (15% and 10%, S4 Table). The atoms involved in direct H-bonding are shown. They have the same color code as the amino acids they belong to. As expected, they belong to residues close by.
(PDF)

S3 Table Same as S2 Table but only for side chains and the occupancy of 25% for the criterion of 60° and 10% for the criterion of 20°. (PDF)

S4 Table Same as S3 Table but only for direct H-bonds and the occupancy of 15% for the criterion of 60° and 10% for the criterion of 20°. (PDF)

S5 Table Helical secondary structure content in the last 110 of standard MD simulations (R#1–3) and for the last 40 ns (R#1 and R#2) and 30 ns (R#3) of the constant pH MD simulations. (PDF)

S1 Text H-Bond networks. (PDF)

## Abbreviations

NPY: Neuropeptide Y
h(p)NPY: human (porcine) NPY
MD simulation: Molecular dynamics simulation
CpHMD simulations: Constant pH molecular dynamics simulations
T-pad: The protein angular dispersion
