# Supplementary material for: pH-dependent structural dynamics of neuropeptide Y in aqueous solution
Source: PLoS One. 2026 Mar 12;21(3):e0343614. doi: 10.1371/journal.pone.0343614 (PMC12981483; doi:10.1371/journal.pone.0343614)
Supplement: S1 File — Here, we present our calculations of the protonation coordinate i, partial charge, and deprotonated fraction Si of residue i, i = 1 for Asp6, i = 2 for Glu10, i = 3 for Asp11, i = 4 for Glu15, i = 5 for Asp16, and i = 6 for His26. (ZIP) [file pone.0343614.s001.zip › S1-S38 Figs.docx]

## Prediction of the pKa values of titratable residues

Here, we present our calculations of the protonation coordinate *i*, partial charge, and deprotonated fraction S*_i_* of residue *i*, *i* = 1 for Asp6, *i* = 2 for Glu10, *i* = 3 for Asp11, *i* = 4 for Glu15, *i* = 5 for Asp16, and *i* = 6 for His26.

**S1 Fig**. **The** $\boldsymbol{\lambda}_{\boldsymbol{1}}$ **coordinate and partial charges of Asp6 during simulated time at pH 7.** (A) Replica 1 (R#1), (B) Replica 2 (R#2) and (C) Replica 3 (R#3).

**S2 Fig**. Same as **S1 Fig**, here calculations were carried out for Asp6 at pH 6.

**S3 Fig**. Same as **S1 Fig**, here for Asp6 at pH 5.

**S4 Fig**. Same as **S1 Fig**, here for Asp6 at pH 4.

**S5 Fig**. Same as **S1 Fig**, here for Asp6 at pH 3.

**S6 Fig**. **Time series of the deprotonated fraction values S of Asp6 at different pH values.**

**S7 Fig**. Same as **S1 Fig**, here for Glu10 at pH 7.

**S8 Fig**. Same as **S1 Fig**, here for Glu10 at pH 6.

**S9 Fig**. Same as **S1 Fig**, here for Glu10 at pH 5.

**S10 Fig**. Same as **S1 Fig**, here for Glu10 at pH 4.

**S11 Fig**. Same as **S1 Fig**, here for Glu10 at pH 3.

**S12 Fig**. Same as **S6 Fig** here for Glu10.

**S13 Fig**. Same as **S1 Fig**, here for Asp11 at pH 7.

**S14 Fig**. Same as **S1 Fig**, here for Asp11 at pH 6.

**S15 Fig**. Same as **S1 Fig**, here for Asp11 at pH 5.

**S16 Fig**. Same as **S1 Fig**, here for Asp11 at pH 4.

**S17 Fig**. Same as **S1 Fig**, here for Asp11 at pH 3.

**S18 Fig.** Same as **S6** **Fig**, calculated for Asp11.

**S19 Fig**. Same as **S1 Fig**, here for Glu15 at pH 7.

**S20 Fig**. Same as **S1 Fig**, here for Glu15 at pH 6.

**S21 Fig**. Same as **S1 Fig**, here for Glu15 at pH 5.

**S22 Fig**. Same as **S1 Fig**, here for Glu15 at pH 4.

**S23 Fig**. Same as **S1 Fig**, here for Glu15 at pH 3.

**S24 Fig**. Same as **S6 Fig**, here for Glu15.

**S25 Fig**. Same as **S1 Fig**, here for Asp16 at pH 7.

**S26 Fig**. Same as **S1 Fig**, here for Asp16 at pH 6.

**S27 Fig**. Same as **S1 Fig**, here for Asp16 at pH 5.

**S28 Fig**. Same as **S1 Fig**, here for Asp16 at pH 4.

**S29 Fig**. Same as **S1 Fig**, here for Asp16 at pH 3.

**S30 Fig**. Same as **S6** **Fig,** here for Asp16.

**S31 Fig**. Same as **S1 Fig**, here for His26 at pH 7.

**S32 Fig**. Same as **S1 Fig**, here for His26 at pH 6.

**S33 Fig**. Same as **S1 Fig**, here for His26 at pH 5.

**S34 Fig**. Same as **S1 Fig**, here for His26 at pH 4.

**S35 Fig**. Same as **S1 Fig**, here for His26 at pH 3.

**S36 Fig**. Same as **S6 Fig**, here for His26.

**S37 Fig**. **Titration curves of titratable residues after 40ns for R#1-2, and 30ns for R#3.**

**S38 Fig.** **Comparison between mean of calculated pK_a_ values of titratable residues and model pK_a_ values.** The model pK_a_ values are taken from the penta-peptide CH_3_CO-AA*X*AA-NH_2_, where *X* represents a titratable amino acid. (A-C) R#1-3.
