# Supplementary material for: pH-dependent structural dynamics of neuropeptide Y in aqueous solution
Source: PLoS One. 2026 Mar 12;21(3):e0343614. doi: 10.1371/journal.pone.0343614 (PMC12981483; doi:10.1371/journal.pone.0343614)
Supplement: S2 File — (ZIP) [file pone.0343614.s002.zip › S39-S63 Figs.docx]

## H-bond networks

**S39 Fig. Intramolecular H-bond networks involving the entire residues (that is, backbone and side chain), averaged over all replica’s simulations at pH 7, using the H-bond angle criteria of 60^0^ or less (A) and of 20^0^ or less (B).** The H-bonds may be either direct or involve 1,2, or 3 water molecules. *Left:* H-bond occupancies (expressed in percentages) shown in a residue heat map.  *Right*: H-bond network. The number of H-bonds (reported either if the occupancy is 50% or more (A) or 10% or more (B)) is shown in purple, whereas the number of water molecules is in orange.

**S40 Fig**. Same as **S39 Fig**, here for pH 6.

**S41 Fig**. Same as **S39 Fig**, here for pH 5.

**S42 Fig**. Same as **S39 Fig**, here for pH 4.

**S43 Fig**. Same as **S39 Fig**, here for pH 3.

**S44 Fig.** Same as **S39 Fig - S43 Fig (A),** *right,* here only for R#1.

**S45 Fig.** Same as **S39 Fig - S43 Fig(A),** *right,* here only for R#2.

**S46 Fig.** Same as **S39 Fig - S43 Fig (A),** *right*, here only for R#3**.**

**S47 Fig.** Same as **S39 Fig - S43 Fig (B),** *right*, here only for R#1.

**S48 Fig.** Same as **S39 Fig - S43 Fig (B),** *right*, here only for R#2.

**S49 Fig.** Same as **S39 Fig - S43 Fig (B),** *right*, here only for R#3.

**S50 Fig.** Same as **S39 Fig,** but only for the side chains, and using an occupancy of 25% for H-bond angle criterion of 60^0^ or less instead of 50% (pH 7). Water mediated and direct H-bonds included.

**S521 Fig.** Same as **S50** **Fig**, here for pH 6.

**S52 Fig.** Same as **S50** **Fig**, here for pH 5.

**S53 Fig.** Same as **S50** **Fig**, here for pH 4.

**S54 Fig.** Same as **S50** **Fig**, here for pH 3.

**S55 Fig.** Same as **S50 Fig - S54 Fig** (A), *right*, here only for R#1, R#2 and R#3.

**S56 Fig.** Same as **S50 Fig - S54 Fig** (B), *right*, here only for R#1, R#2 and R#3.

**S57 Fig.** Same as **S50 Fig**. except that only direct H-bonds are considered and an occupancy of 15% is set for the criterion of H-bond angle of 60^0^. The pH is 7.

**S58 Fig.** Same as **S57** **Fig**, here for pH 6.

**S59 Fig.** Same as **S57** **Fig**, here for pH 5.

**S60 Fig.** Same as **S57** **Fig**, here for pH 4.

**S61 Fig.** Same as **S57** **Fig**, here for pH 3.

**S62 Fig**. Same as **S57 Fig - S61 Fig** (A), *right*, here only for R#1, R#2 and R#3.

**S63 Fig**. Same as **S57 Fig - S61 Fig** (B), *right*, here only for R#1, R#2 and R#3, except for pH 3, where the minimum occupancy is 6%.
