# Supplementary material for: pH-dependent structural dynamics of neuropeptide Y in aqueous solution
Source: PLoS One. 2026 Mar 12;21(3):e0343614. doi: 10.1371/journal.pone.0343614 (PMC12981483; doi:10.1371/journal.pone.0343614)
Supplement: S3 File — Standard MD simulations. Here all the titratable side chains were in their standard protonation states, as chosen at the beginning of the simulation. During the final 110 ns of each of the three MD simulations (lasting 500 ns), the α-helical segment (Glu15–Ile31), was conserved whereas the N- and C-terminal regions were rather disordered. A summary of the three analyses is offered in S5 Table and S64–S69 Figs in S3 File. (ZIP) [file pone.0343614.s003.zip › S64-S75 Figs.docx]

**3. NPY conformation dynamics**

Standard MD simulations. Here all the titratable side chains were in their standard protonation states, as chosen at the beginning of the simulation. During the final 110 ns of each of the three MD simulations (lasting 500 ns), the α-helical segment (Glu15–Ile31), was conserved whereas the N- and C-terminal regions were rather disordered. A summary of the three analyses is offered in **S5 Table** and **S64–S69 Fig**s.

**S64 Fig**. Secondary structure content of pNPY, plotted as a function of time, in the three standard MD simulations, for the last 110 ns (A: R#1, B: R#2, C: R#3). The $\alpha$-helix is colored in magenta, turn in teal the extended configuration in yellow, 3-10 helix in blue and coil in white. Figure prepared with VMD(34).

**S65 Fig**. Same as **S64 Fig** but for constant pH simulations at pH 7, for all the timescale explored in replicas R#1-3.

**S66 Fig**. Same as **S64 Fig**, here for pH 6.

**S67 Fig**. Same as **S64 Fig**, here for pH 5.

**S68 Fig**. Same as **S64 Fig**, here for pH 4.

**S69 Fig**. Same as **S64 Fig**, here for pH 3.

**S70 Fig. Top:** Fluctuations as backbone as quantified by PAD(60) values, calculated for CpHMD simulations at pH 7 and pH 6. Bottom: Bottom: mean PAD values for the 3 replicas and standard deviation.

**S71 Fig.** Same as **S70 Fig**, here for CpHMD simulations at pH 6 and pH 5.

**S72 Fig.** Same as **S70 Fig**, here for CpHMD simulations at pH 5 and pH 4.

**S73 Fig.** Same as **S70 Fig**, here for CpHMD simulations at pH 4 and pH 3.
