# Supplementary material for: pH-dependent structural dynamics of neuropeptide Y in aqueous solution
Source: PLoS One. 2026 Mar 12;21(3):e0343614. doi: 10.1371/journal.pone.0343614 (PMC12981483; doi:10.1371/journal.pone.0343614)
Supplement: S1 Table — (PDF) [file pone.0343614.s004.pdf]

pH-dependent structural dynamics of neuropeptide Y in aqueous solution

Hoa Thi Nguyen,[1,2] *Marc Spehr,*[2,3] *Ana-Nicoleta Bondar,*[1,4*] *Paolo Carloni*[1,2,5*]

[1]Forschungszentrum Jülich, Computational Biomedicine, INM-9, Wilhelm-Johnen Straße, 52428 Jülich, Germany

[2]Research Training Group 2416 MultiSenses – MultiScales, RWTH Aachen University, 52074 Aachen, Germany

[3]RWTH Aachen University, Institute for Biology II, Department of Chemosensation, Worringerweg 3, D-52074 Aachen, Germany

[4]University of Bucharest, Faculty of Physics, Atomistilor 405, Magurele, Romania

[5]RWTH Aachen University, Molecular Science and Engineering, Aachen, Germany

*Correspondent authors

Supporting Information

Supporting Information Tables

S1 Table. Mean pK_a values obtained from our simulations.

	Model pK_a	R#1		R#2		R#3	
		pK_a	ΔpK_a	pK_a	ΔpK_a	pK_a	ΔpK_a
Asp6	4.0	4.5	0.5	4.5	0.5	4.5	0.5
Glu10	4.4	3.6	-0.8	3.7	-0.7	3.5	-0.9
Asp11	4.0	5.3	1.3	5.1	1.1	5.3	1.3
Glu15	4.4	3.5	-0.9	3.7	-0.7	3.7	-0.7
Asp16	4.0	2.9	-1.1	3.2	-0.8	2.4	-1.6
His26	7.0	6.3	-0.7	6.4	-0.6	6.4	-0.6
