# Supplementary material for: pH-dependent structural dynamics of neuropeptide Y in aqueous solution
Source: PLoS One. 2026 Mar 12;21(3):e0343614. doi: 10.1371/journal.pone.0343614 (PMC12981483; doi:10.1371/journal.pone.0343614)
Supplement: S2 Table — This and the next two tables have as occupancies as those in S39–S49 Figs in S2 File (50% and 10% for criteria of 60° and 20°, respectively), S50–S56 in S2 File (25% and 10%, S3 Table), and S57–S63 in S2 File (15% and 10%, S4 Table). The atoms involved in direct H-bonding are shown. They have the same color code as the amino acids they belong to. As expected, they belong to residues close by. (PDF) [file pone.0343614.s005.pdf]

pH-dependent structural dynamics of neuropeptide Y in aqueous solution

Hoa Thi Nguyen,[1,2] Marc Spehr,[2,3] Ana-Nicoleta Bondar,[1,4] Paolo Carloni[1,2,5*]*

[1]Forschungszentrum Jülich, Computational Biomedicine, INM-9, Wilhelm-Johnen Straße, 52428 Jülich, Germany

[2]Research Training Group 2416 MultiSenses – MultiScales, RWTH Aachen University, 52074 Aachen, Germany

[3]RWTH Aachen University, Institute for Biology II, Department of Chemosensation, Worringerweg 3, D-52074 Aachen, Germany

[4]University of Bucharest, Faculty of Physics, Atomistilor 405, Magurele, Romania

[5]RWTH Aachen University, Molecular Science and Engineering, Aachen, Germany

*Correspondent authors

Supporting Information

Supporting Information Tables

S2 Table. Occupancies of either direct H-bonds or water-mediated bridges among entire residues (that is, backbone and side chains), averaged over the constant pH simulations at different pH values. This and the next two tables report the occupancies as set in **S39-S49 Figs** (50% and 10% for criteria of 60^0 and 20^0, respectively), S50-S56 (25 % and 10%, **Table S3**), and S57-S63 (15% and 10%, **Table S4**). The atoms involved in direct H-bonding are shown. They have the same color code as the amino acids they belong to. As expected, they belong to residues close by.

pH	H-bond	R#1		R#2		R#3	
		60^0	20^0	60^0	20^0	60^0	20^0
pH7	D6-S3	96-0.1 N-O/OG, OD1/OD2-OG/N	42-0.3 N-O, OD1/OD2-OG/N	95-0.1 N-O/OG, O/OD1/OD2-OG	45-0.1 N-O, OD1/OD2-OG	96-0.1 N-O/OG, OD1/OD2-OG/N	46-0.2 N-O, OD1/OD2-OG
	E10-P8	No	No	No	No	51-1.4 (only water-mediated)	No
	E10-D11	63-1.4 OE1/OE2-N	17-1.4 OE2-N	50-1.7 OE1/OE2-N	10-1.6 (only water-mediated)	83-1.2 OE1/OE2-N	29-1.2 OE1/OE2-N
	E10-Y21	No	No	No	No	70-1.2 OE1/OE2-OH	24-0.7 OE1/OE2-OH
	E10-R25	No	No	No	No	75-0.8 OE1/OE2-NH1/NH2	40-0.3 OE1/OE2-NH1/NH2
	D11-K4	No	10-0.1 OD1/OD2-NZ	No	No	No	No
	D11-A12	52-1.4 OD1/OD2-N	No	No	No	66-1.9 (only water-mediated)	11-2.1 (only water-mediated)
	D11-R25	No	No	73-0.3 OD1/OD2-NH1/NH2	52-0.0 OD1/OD2-NH1/NH2	No	No
	E15-D16	No	No	No	No	56-1.9 (only water-mediated)	12-1.6 (only water-mediated)
	E15-R19	95-0.1 O-N/NH2, OE1/OE2-NE/NH1/NH2	49-0.1 O-N, OE1/OE2-NE/NH1/NH2	97-0.0 O-N, OE1/OE2-NE/NH1/NH2	50-0.1 O-N, OE1/OE2-NE/NH1/NH2	99-0.0 O-N, OE1/OE2-NE/NH1/NH2	60-0.1 O-N, OE1/OE2-NH1/NH2

	D16-R19	74-0.4 O-N, OD1/OD2-NE/NH1/NH2	31-0.3 OD1-NE/NH1/NH2, OD2-NH1/NH2	70-0.3 O-N, OD1/OD2-NE/NH1/NH2	32-0.2 OD1/OD2-NH1/NH2	84-0.4 O-N, OD1-NE/NH1/NH2, OD2-NH1/NH2	29-0.3 OD1/OD2-NH1/NH2
	D16-Y20	87-0.0 O-N OD2-OH	21-0.1 O-N	83-0.0 O-N	21-0.0 O-N	91-0.0 O-N	24-0.0 O-N
	H26-S22	94-0.1 ND1/N-O	32-0.1 ND1/N-O	92-0.1 ND1/N-O, NE2-OG	27-0.1 ND1/N-O	84-0.3 ND1/N-O, NE2-OG	26-0.1 ND1/N-O
	H26-N29	55-1.2 O-N, ND1-O/ND2	No	54-0.7 O-N/ND2	No	52-1.3 O-N	No
	H26-L30	77-0.1 O-N	17-0.0 O-N	71-0.0 O-N	15-0.0 O-N	51-0.1 O-N	No
pH6	D6 - S3	98-0.1 N-O/OG, OD1/OD2-N/OG	32-0.4 N-O, OD1/OD2-N/OG	94-0.1 N-O/OG, OD1-OG, OD2-N/OG	54-0.1 N-O/OG, OD1/OD2-OG	96-0.1 N-O, OD1/OD2-N/OG	52-0.2 N-O/OG, OD1/OD2-OG
	E10-N7	No	No	No	12-0.3 OE1-ND2, N-O/OD1	No	No
	E10-D11	79-0.1 OE1/OE2-N	26-1.3 (only water-mediated)	53-1.3 OE1/OE2-N	14-1.4 (only water-mediated)	84-0.9 OE1/OE2-N	34-0.8 OE1/OE2-N
	E10-Y21	63-1.4 OE1/OE2-OH	19-0.6 OE1/OE2-OH	No	No	64-1.2 OE1/OE2-OH	20-0.6 OE1/OE2-OH
	E10-R25	74-0.7 OE1/OE2-NH1/NH2	36-0.2 OE1/OE2-NH1/NH2	No	No	80-0.5 OE1/OE2-NE/NH1/NH2	45-0.1 OE1/OE2-NH1/NH2, OE2-NE
	D11-A12	61-1.9 (only water-mediated)	No	No	No	54-1.9 (only water-mediated)	No
	D11-R25	No	No	No	17-0.2 OD1/OD2-NH1/NH2	No	No
	E15-D16	54-2.0 (only water-mediated)	No	No	No	58-2.0 (only water-mediated)	12-1.9 (only water-mediated)
	E15-R19	99-0.0 O-N, OE1/OE2-NE/HN1/NH2	51-0.1 O-N, OE1/OE2-NE/HN1/NH2	94-0.1 O-N, OE1/OE2-NE/HN1/NH2	39-0.2 O-N, OE1/OE2-NE/HN1/NH2	98-0.0 O-N, OE1/OE2-HN1/NH2	49-0.1 O-N, OE1/OE2-HN1/NH2
	D16-R19	64-0.0 O-N,	15-0.5	85-0.2 O-N,	41-0.2	97-0.1 O-N/NE,	58-0.1

		OD1-NE/NH1/NH2, OD2-NH1/NH2	OD1/OD2-NH1/NH2	OD1/OD2-NE/NH1/NH2	OD1/OD2-NH1/NH2	OD1/OD2-NE/NH1/NH2	OD1/OD2-NE/NH1/NH2
	D16-Y20	89-0.0 O-N	24-0.0 O-N	84-0.1 O-N	32-0.1 O-N	94-0.0 O-N, OD1-OH	38-0.1 O-N
	H26-S22	93-0.1 N/ND1-O, NE2-OG	34-0.1 N/ND1-O	94-0.0 N/ND1-O, NE2-OG	34-0.0 N/ND1-O	92-0.1 N/ND1-O, NE2-OG	31-0.1 N-O
	H26-N29	53-1.0 O-N, ND1-OD1	No	No	No	No	No
	H26-L30	82-0.0 O-N	18-0.0 O-N	69-0.0 O-N	21-0.0 O-N	85-0.0 O-N	20-0.0 O-N
pH5	D6-S3	95-0.1 N-O/OG, OD1/OD2-OG	29-0.2 N-O, OD1/OD2-OG	94-0.1 N-O/OG, OD1/OD2-N/OG	48-0.1 N-O/OG, OD1/OD2-N/OG	93-0.1 N-O/OG, OD1/OD2-OG	37-0.2 N-O, OD1/OD2-OG
	E10-P8	52-1.4 (only water-mediated)	No	No	No	No	No
	E10-D11	71-1.5 OE1/OE2-N	17-1.3 OE1/OE2-N	54-1.2 OE1/OE2-N	18-1.2 OE2-N	59-1.6 OE2-N	13-1.5 (only water-mediated)
	E10-Y21	67-1.3 OE1/OE2-OH	20-0.8 OE1/OE2-OH	No	No	58-1.5 OE1/OE2-OH	16-1.0 OE1/OE2-OH
	E10-R25	No	17-0.3 OE1/OE2-NH1/NH2	67-0.4 O-NH1, OE1/OE2-NE/NH1/NH2	34-0.1 OE1/OE2-NE/NH1/NH2	No	No
	D11-A12	55-1.9 (only water-mediated)	No	No	No	No	No
	E15-R19	99-0.0 O-N, OE1/OE2-NE/NH1/NH2	53-0.1 O-N, OE1/OE2-NE/NH1/NH2	95-0.1 O-N, OE1/OE2-NE/NH1/NH2	43-0.1 O-N, OE1/OE2-NE/NH1/NH2	98-0.0 O-N, OE1/OE2-NE/NH1/NH2	46-0.1 O-N, OE1/OE2-NH1/NH2
	D16-R19	82-0.4 O-N, OD1/OD2-NE/NH1/NH2	38-0.2 OD1-NH1/NH2, OD2-NE/NH1/NH2	76-0.3 O-N, OD1/OD2-NE/NH1/NH2	32-0.1 OD1/OD2-NE/NH1/NH2	83-0.3 O-N, OD1/OD2-NE/NH1/NH2	41-0.1 OD1-NH1/NH2, OD2-NE/NH1/NH2
	D16-Y20	95-0.0 O-N	24-0.0 O-N	84-0.1 O-N	28-0.1 O-N	90-0.0 O-N	22-0.0 O-N
	H26-S22	89-0.1 N/ND1-O, NE2-OG	30-0 N/ND1-O	94-0.0 N/ND1-O, NE2-OG	34-0.0 N-O	91-0.0 N/ND1-O, NE2-OG	17-0.1 N/ND1-O

	H26-N29	53-0.8 O-N/ND2	No	No	No	No	No
	H26-L30	85-0.0 O-N	22-0.0 O-N	83-0.0 O-N	17-0.0 N-O	No	No
pH4	D6-S3	92-0.1 N-O/OG, OD1/OD2-N/OG	27-0.2 N-O, OD1/OD2-N/OG	96-0.1 N-O, OD1/OD2-OG	19-0.2 N-O, OD1/OD2-OG	95-0.1 N-O, OD1/OD2-N/OG	25-0.3 N-O, OD1-OG, OD2-N/OG
	E10-D11	59-1.5 OE1/OE2-N	12-1.4 OE1-N	55-1.5 OE1/OE2-N	12-1.3 OE1-N	58-1.7 (only water-mediated)	No
	E10-Y21	60-1.1 OE1/OE2-OH	20-0.5 OE1/OE2-OH	51-1.5 OE1/OE2-OH	13-0.7 OE1/OE2-OH	59-1.5 OE1/OE2-OH	15-0.9 OE1/OE2-OH
	E10-R25	No	11-0.7 OE1/OE2-NH1/NH2	No	21-0.2 OE1/OE2-NH1/NH2	No	No
	E15-R19	97-0.0 O-N, OE1/OE2-NE/NH1/NH2	49-0.1 O-N, OE1/OE2-NE/NH1/NH2	98-0.0 O-N, OE1/OE2-NH1/NH2	42-0.1 OE1/OE2-NH1/NH2	95-0.0 O-N/NE, OE1/OE2-NH1/NH2	34-0.1 O-N, OE1/OE2-NH1/NH2
	D16-R19	81-0.4 O-N, OD1/OD2-NE/NH1/NH2	32-0.2 OD1/OD2-NE/NH1/NH2	85-0.2 O-N, OD1/OD2-NE/NH1/NH2	43-0.1 OD1/OD2-NE/NH1/NH2	83-0.3 O-N, OD1/OD2-NE/NH1/NH2	37-0.1 O-N, OD1/OD2-NE/NH1/NH2
	D16-Y20	89-0.0 O-N	23-0.0 O-N	90-0.0 O-N	25-0.0 O-N	89-0.0 O-N	29-0.0 O-N
	H26-S22	92-0.0 N/ND1-O, NE2-OG	30-0.1 N/ND1-O	87-0.1 N/ND1-O, NE2-OG	24-0.1 N/ND1-O	97-0.0 N/ND1-O, NE2-OG	35-0.1 N/ND1-O
	H26-N29	63-0.4 O-N/ND2	No	No	No	53-0.7 O-N/ND2	No
	H26-L30	58-0.0 O-N	No	71-0.0 O-N	14-0.0 O-N	79-0.0 O-N	18-0.1 O-N
pH3	D6-S3	90-0.2 N-O, OD1/OD2-N/OG	20-0.3 N-O, OD1/OD2-OG	97-0.1 N-O, OD1/OD2-N/OG	26-0.2 N-O, OD1/OD2-N/OG	99-0.0 N-O	13-0.4 N-O
	E10-G9	No	No	53-1.4 OE1-N	No	No	No
	E10-D11	52-1.7 OE1/OE2-N	No	57-1.9 OE1/OE2-N	No	57-1.5 OE1/OE2-N	11-1.3 OE1-N
	E10-R25	No	No	No	No	53-0.8 OE1/OE2-NH1/NH2	24-0.0 OE1/OE2-NH1/NH2
	E15-D16	No	No	52-1.9 (only water-mediated)	No	No	No

	E15-R19	97-0.1 O-N, OE1/OE2-NE/NH1/NH2	47-0.1 O-N, OE1/OE2-NH1/NH2	90-0.1 (only water-mediated)	38-0.1 O-N, OE1-NE/NH1 OE2-NH1/NH2	98-0.0 O-N, OE1/OE2-NH1/NH2	42-0.0 O-N, OE2-NH1
	D16-R19	73-0.6 O-N, OD1/OD2-NE/NH1/NH2	22-0.3 O-N, OD1/OD2-NH1/NH2	63-0.9 O-N, OD1/OD2-NE/NH1/NH2	11-0.6 OD1/OD2-NH1/NH2	76-0.7 O-N, OD1/OD2-NE/NH1/NH2	29-0.0 OD1/OD2-NE/NH1/NH2
	D16-Y20	88-0.0 O-N	20-0.0 O-N	85-0.1 O-N, OD1/OD2-OH	26-0.0 O-N	93-0.0 O-N	26-0.0 O-N
	H26-S22	91-0.0 N-O	27-0.1 N-O	95-0.1 N/ND1-O, NE2-OG	31-0.1 N/ND1-O	95-0.1 N/ND1-O, NE2-OG	27-0.0 N/ND1-O
	H26-N29	No	No	51-0.9 O-N	No	No	No
	H26-L30	80-0.0 O-N	19-0.0 O-N	69-0.0 O-N	10-0.0 O-N	72-0.0 O-N	17-0.0 O-N
