# Supplementary material for: pH-dependent structural dynamics of neuropeptide Y in aqueous solution
Source: PLoS One. 2026 Mar 12;21(3):e0343614. doi: 10.1371/journal.pone.0343614 (PMC12981483; doi:10.1371/journal.pone.0343614)
Supplement: S3 Table — (PDF) [file pone.0343614.s006.pdf]

pH-dependent structural dynamics of neuropeptide Y in aqueous solution

Hoa Thi Nguyen,[1,2] *Marc Spehr,*[2,3] *Ana-Nicoleta Bondar,*[1,4*] *Paolo Carloni*[1,2,5*]

[1]Forschungszentrum Jülich, Computational Biomedicine, INM-9, Wilhelm-Johnen Straße, 52428 Jülich, Germany

[2]Research Training Group 2416 MultiSenses – MultiScales, RWTH Aachen University, 52074 Aachen, Germany

[3]RWTH Aachen University, Institute for Biology II, Department of Chemosensation, Worringerweg 3, D-52074 Aachen, Germany

[4]University of Bucharest, Faculty of Physics, Atomistilor 405, Magurele, Romania

[5]RWTH Aachen University, Molecular Science and Engineering, Aachen, Germany

*Correspondent authors

Supporting Information

Supporting Information Tables

S3 Table. Same as **S2** but only for side chains and the occupancy of 25% for the criterion of 60^0 and 10% for the criterion of 20^0.

pH	H-bond criterion	R#1		R#2		R#3	
		60^0	20^0	60^0	20^0	60^0	20^0
pH7	D6-S3	58-1.0 OD1/OD2-OG	26-0.3 OD1/OD2-OG	67-0.4 OD1/OD2-OG	40-0.1 OD1/OD2-OG	74-0.6 OD1/OD2-OG	38-0.2 OD1/OD2-OG
	D6-Y20	No	No	No	No	35-1.9 (only water-mediated)	No
	E10-D11	35-1.9 (only water-mediated)	No	29-2.0 (only water-mediated)	No	61-1.6 (only water-mediated)	18-1.4 (only water-mediated)
	E10-Y21	No	No	No	No	60-1.1 OE1/OE2-OH	22-0.6 OE1/OE2-OH
	E10-R25	No	No	26-1.0 (only water-mediated)	No	75-0.8 OE1/OE2-NH1/NH2	40-0.3 OE1/OE2-NH1/NH2
	D11-K4	No	10-0.1 (only water-mediated)	No	No	No	No
	D11-R25	No	No	70-0.3 OD1/OD2-NH1/NH2	52-0.0 OD1/OD2-NH1/NH2	No	No
	E15-D16	31-2.0 (only water-mediated)	No	No	No	47-1.9 (only water-mediated)	10-1.5 (only water-mediated)
	E15-R19	64-0.6 OE1/OE2-NE/NH1/NH2	26-0.3 OE1/OE2-NE/NH1/NH2	58-0.7 OE1/OE2-NE/NH1/NH2	22-0.3 OE1/OE2-NE/NH1/NH2	83-0.4 OE1/OE2-NE/NH1/NH2	40-0.2 OE1/OE2-NH1/NH2
	D16-R19	67-0.5 OD1/OD2-NE/NH1/NH2	30-0.3 OD1-NE/NH1/NH2,	65-0.9 OD1/OD2-NE/NH1/NH2	32-0.2 OD1/OD2-NH1/NH2	79-0.4 OD1/OD2-NE/NH1/NH2	29-0.2 OD1/OD2-NH1/NH2

			OD2-NH1/NH2				
	H26-S22	No	No	No	No	31-2.0 (only water-mediated)	No
	H26-N29	32-1.7 (only water-mediated)	No	25-1.7 NE2-ND2	No	No	No
pH6	D6-S3	48-1.2 OD1/OD2-OG	17-0.3 OD1/OD2-OG	76-0.2 OD1/OD2-OG	49-0.1 OD1/OD2-OG	74-0.4 OD1/OD2-OG	44-0.1 OD1/OD2-OG
	D6-Y20	33-2.0 (only water-mediated	No	No	No	No	No
	E10-D11	61-1.7 (only water-mediated)	17-1.4 (only water-mediated)	56-1.7 (only water-mediated)	15-1.4 (only water-mediated)	29-1.9 (only water-mediated)	No
	E10-Y21	53-1.3 (only water-mediated)	17-0.5 (only water-mediated)	54-1.2 (only water-mediated)	19-0.6 (only water-mediated)	No	No
	E10-R25	74-0.6 OE1/OE2-NH1/NH2	36-0.2 OE1/OE2-NH1/NH2	80-0.5 OE1/OE2-NE/NH1/NH2	45-0.1 OE1-NH1/NH2, OE2-NE/NH1/NH2	25-1.2 (only water-mediated)	No
	D11-R25	No	No	No	No	No	16-0.2 OD1/OD2-NH1/NH2
	E15-D16	33-2.3 (only water-mediated)	No	28-2.0 (only water-mediated)	No	47-2.1 (only water-mediated)	No
	E15-R19	67-0.9 OE1/OE2-NE/NH1/NH2	23-0.4 (only water-mediated)	51-1.0 OE1/OE2-NE/NH1/NH2	18-0.4 OE1/OE2-NE/NH1/NH2	70-0.6 OE1/OE2-NH1/NH2	30-0.2 OE1/OE2-NH1/NH2
	D16-R19	54-1.1 OD1-NE/NH1/NH2, OD2-NH1/NH2	14-0.4 (only water-mediated)	81-0.3 OD1/OD2-NE/NH1/NH2	40-0.1 OD1/OD2-NH1/NH2	95-0.1 OD1/OD2-NE/NH1/NH2	57-0.1 OD1/OD2-NE/NH1/NH2
	H26-S22	34-1.9	No	26-2.0	No	No	No

		(only water-mediated)		(only water-mediated)			
	H26-N29	33-1.6 (only water-mediated)	No	No	No	30-1.8 (only water-mediated)	No
pH5	D6-S3	54-1.1 OD1/OD2-OG	20-0.3 OD1/OD2-OG	67-0.7 OD1/OD2-OG	31-0.2 OD1/OD2-OG	45-0.6 OD1/OD2-OG	25-0.1 OD1/OD2-OG
	D6-Y20	34-2.0 (only water-mediated)	No	34-1.8 (only water-mediated)	No	No	No
	E10-D11	53-1.8 (only water-mediated)	10-1.5 (only water-mediated)	45-1.6 (only water-mediated)	No	31-1.9 (only water-mediated)	No
	E10-Y21	58-1.3 OE1/OE2-OH	18-0.6 (only water-mediated)	53-1.5 (only water-mediated)	14-0.8 (only water-mediated)	No	No
	E10-R25	47-1.2 OE1/OE2-NH1/NH2	17-0.3 OE1/OE2-NH1/NH2	27-2.1 (only water-mediated)	No	65-0.4 OE1/OE2-NE/NH1/NH2	34-0.1 OE1/OE2-NH1/NH2
	D11-R25	No	No	No	No	No	13-0.1 OD1/OD2-NH1/NH2
	E15-D16	34-2.0 (only water-mediated)	No	30-2.2 (only water-mediated)	No	28-2.1 (only water-mediated)	No
	E15-R19	56-0.7 OE1/OE2-NE/NH1/NH2	26-0.2 OE1/OE2-NE/NH1/NH2	55-1.0 OE1/OE2-NE/NH1/NH2	16-0.4 OE1/OE2-NE/NH1/NH2	57-0.8 OE1/OE2-NE/NH1/NH2	21-0.3 OE1/OE2-NE/NH1/NH2
	D16-R19	78-0.5 OD1/OD2-NE/NH1/NH2	37-0.2 OD1-NH1/NH2, OD1/OD2-NE/NH1/NH2	77-0.3 OD1/OD2-NE/NH1/NH2	40-0.1 OD1-NH1/NH2, OD1/OD2-NE/NH1/NH2	69-0.4 OD1/OD2-NE/NH1/NH2	32-0.1 OD1/OD2-NE/NH1/NH2
	H26-S22	31-2.0 (only water-mediated)	No	No	No	No	No
	H26-N29	33-1.6	No	No	No	27-1.8	No

		(only water-mediated)				(only water-mediated)	
pH4	D6-S3	45-1.2 OD1/OD2-OG	14-0.3 OD1/OD2-OG	48-1.4 OD1/OD2-OG	12-0.4 (only water-mediated)	33-1.6 (only water-mediated)	No
	E10-D11	42-1.8 (only water-mediated)	No	47-1.8(only water-mediated)	No	37-1.8 (only water-mediated)	No
	E10-Y21	54-1.0 OE1/OE2-OH	19-0.4 OE1/OE2-OH	51-1.5 (only water-mediated)	14-0.8 (only water-mediated)	42-1.4 (only water-mediated)	12-0.6 (only water-mediated)
	E10-R25	46-1.5 (only water-mediated)	11-0.7 (only water-mediated)	37-1.8 (only water-mediated)	No	45-0.9 OE1/OE2-NH1/NH2	21-0.2 OE1/OE2-NH1/NH2
	E15-D16	28-2.1 (only water-mediated)	No	26-2.1 (only water-mediated)	No	25-2.2 (only water-mediated)	No
	E15-R19	50-1.1 OE1/OE2-NE/NH1/NH2	15-0.4 OE1/OE2-NE/NH1/NH2	38-1.4 OE1/OE2-NE/NH1/NH2	No	34-1.4 (only water-mediated)	No
	D16-R19	77-0.4 OD1/OD2-NE/NH1/NH2	32-0.2 OD1/OD2-NE/NH1/NH2	78-0.3 OD1/OD2-NE/NH1/NH2	37-0.1 OD1/OD2-NE/NH1/NH2	80-0.3 OD1/OD2-NE/NH1/NH2	42-0.1 OD1/OD2-NE/NH1/NH2
	H26-S22	No	No	35-1.9 (only water-mediated)	No	No	No
	H26-N29	No	No	29-1.7 (only water-mediated)	No	25-1.6 (only water-mediated)	No
pH3	D6-S3	No	No	No	No	40-1.5	No
	E10-D11	31-2.0 (only water-mediated)	No	35-1.9 (only water-mediated)	No	37-2.0 (only water-mediated)	No
	E10-Y21	No	No	30-1.6	No	No	No

				(only water-mediated)			
	E10-R25	28-1.2 (only water-mediated)	No	53-0.8 OE1/OE2-NH1/NH2	24-0.1 OE1/OE2-NH1/NH2	No	No
	E15-R19	49-1.1 OE1/OE2-NE/NH1/NH2	11-0.4 (only water-mediated)	31-1.7 (only water-mediated)	No	43-1.5 (only water-mediated)	No
	D16-R19	65-0.7 OD1/OD2-NE/NH1/NH2	21-0.3 OD1/OD2-NH1/NH2	47-1.2 OD1/OD2-NE/NH1/NH2	10-0.6 OD1/OD2-NH1/NH2	68-0.7 OD1/OD2-NE/NH1/NH2	28-0.2 OD1/OD2-NE/NH1/NH2
	H26-S22	No	No	33-1.8 (only water-mediated)	No	No	No
	H26-N29	No	No	27-1.7 (only water-mediated)	No	No	No
