# Supplementary material for: pH-dependent structural dynamics of neuropeptide Y in aqueous solution
Source: PLoS One. 2026 Mar 12;21(3):e0343614. doi: 10.1371/journal.pone.0343614 (PMC12981483; doi:10.1371/journal.pone.0343614)
Supplement: S4 Table — (PDF) [file pone.0343614.s007.pdf]

pH-dependent structural dynamics of neuropeptide Y in aqueous solution

Hoa Thi Nguyen,[1,2] *Marc Spehr,*[2,3] *Ana-Nicoleta Bondar,*[1,4*] *Paolo Carloni*[1,2,5*]

[1]Forschungszentrum Jülich, Computational Biomedicine, INM-9, Wilhelm-Johnen Straße, 52428 Jülich, Germany

[2]Research Training Group 2416 MultiSenses – MultiScales, RWTH Aachen University, 52074 Aachen, Germany

[3]RWTH Aachen University, Institute for Biology II, Department of Chemosensation, Worringerweg 3, D-52074 Aachen, Germany

[4]University of Bucharest, Faculty of Physics, Atomistilor 405, Magurele, Romania

[5]RWTH Aachen University, Molecular Science and Engineering, Aachen, Germany

*Correspondent authors

Supporting Information

Supporting Information Tables

S4 Table. Same as **S3** but only for direct H-bonds and the occupancy of 15% for the criterion of 60^0 and 10% for the criterion of 20^0.

pH	H-bond criterion	R#1		R#2		R#3	
		60^0	20^0	60^0	20^0	60^0	20^0
pH7	D6-S3	30 OD1/OD2-OG	21 OD1/OD2-OG	52 OD1/OD2-OG	37 OD1/OD2-OG	49 OD1/OD2-OG	32 OD1/OD2-OG
	E10-Y21	No	No	No	No	19 OE1/OE2-OH	12 OE1/OE2-OH
	E10-R25	No	No	No	No	43 OE1/OE2-NH1/NH2	33 OE1/OE2-NH1/NH2
	D11-K4	20 OD2-NZ	No	No	No	No	No
	D11-R25	No	No	61 OD1/OD2-NH1/NH2	51 OD1/OD2-NH1/NH2	No	No
	E15-R19	38 OE1/OE2-NE/NH1/NH2	21 OE1/OE2-NE/NH1/NH2	34 OE1/OE2-NE/NH1/NH2	17 OE1/OE2-NE/NH1/NH2	63 OE1/OE2-NE/NH1/NH2	35 OE1/OE2-NH1/NH2
	D16-R19	45 OD1/OD2-NE/NH1/NH2	24 OD1-NE/NH1/NH2, OD2-NH1/NH2	51 OD1/OD2-NE/NH1/NH2	28 OD1/OD2-NH1/NH2	54 OD1/OD2-NE/NH1/NH2	23 OD1/OD2-NH1/NH2
pH6	D6-S3	20 OD1/OD2-OG	14 OD1/OD2-OG	67 OD1/OD2-OG	47 OD1/OD2-OG	69 OD1/OD2-OG	41 OD1/OD2-OG
	E10-Y21	16 OE1/OE2-OH	12 OE1/OE2-OH	No	No	15 OE1/OE2-OH	10 OE1/OE2-OH
	E10-R25	48 OE1/OE2-NH1/NH2	32 OE1/OE2-NH1/NH2	No	No	59 OE1/OE2-NE/NH1/NH2	42 OE1-NH1/NH2, OE2-NE/NH1/NH2
	D11-R25	No	No	18 OD1/OD2-NH1/NH2	14 OD1/OD2-NH1/NH2	No	No
	E15-R19	34 OE1/OE2-NE/NH1/NH2	No	23 OE1/OE2-NE/NH1/NH2	13 OE1/OE2-NE/NH1/NH2	44 OE1/OE2-NH1/NH2	25 OE1/OE2-NH1/NH2occu
	D16-R19	24 OD1-NE/NH1/NH2,	No	67 OD1/OD2-NE/NH1/NH2	35 OD1/OD2-NE/NH1/NH2	89 OD1/OD2-NE/NH1/NH2	53 OD1/OD2-NH1/NH2

		OD2-NH1/NH2					
pH5	D6-S3	23 OD1/OD2-OG	15 OD1/OD2-OG	33 OD1/OD2-OG	23 OD1/OD2-OG	42 OD1/OD2-OG	26 OD1/OD2-OG
	E10-Y21	16 OE1/OE2-OH	No	No	No	No	No
	E10-R25	19 OE1/OE2-NH1/NH2	14 OE1/OE2-NH1/NH2	51 OE1/OE2-NE/NH1/NH2	32 OE1/OE2-NH1/NH2	No	No
	D11-R25	No	No	No	13 OD1/OD2-NH1/NH2	No	No
	E15-R19	36 OE1/OE2-NE/NH1/NH2	22 OE1/OE2-NE/NH1/NH2	32 OE1/OE2-NE/NH1/NH2	17 OE1/OE2-NE/NH1/NH2	24 OE1/OE2-NE/NH1/NH2	11 OE1/OE2-NE/NH1/NH2
	D16-R19	55 OD1/OD2-NE/NH1/NH2	31 OD1-NH1/NH2, OD2-NE/NH1/NH2	54 OD1/OD2-NE/NH1/NH2	29 OD1/OD2-NE/NH1/NH2	66 OD1/OD2-NE/NH1/NH2	37 OD1-NH1/NH2, OD2-NE/NH1/NH2
pH4	D6-S3	20 OD1/OD2-OG	11 OD1/OD2-OG	No	No	16 OD1/OD2-OG	No
	E10-Y21	23 OE1/OE2-OH	14 OE1/OE2-OH	No	No	No	No
	E10-R25	No	No	24 OE1/OE2-NH1/NH2	18 OE1/OE2-NH1/NH2	No	No
	E15-R19	20 OE1/OE2-NE/NH1/NH2	10 OE1/OE2-NE/NH1/NH2	No	No	No	No
	D16-R19	55 OD1/OD2-NE/NH1/NH2	27 OD1/OD2-NE/NH1/NH2	68 OD1/OD2-NE/NH1/NH2	38 OD1/OD2-NE/NH1/NH2	65 OD1/OD2-NE/NH1/NH2	34 OD1/OD2-NE/NH1/NH2
pH3	E10-R25	No	No	No	No	30 OE1/OE2-NH1/NH2	21 OE1/OE2-NH1/NH2
	E15-R19	20 OE1/OE2-NE/NH1/NH2	No	No	No	No	No
	D16-R19	39 OD1/OD2-NE/NH1/NH2	17 OD1/OD2-NH1/NH2	16 OD1/OD2-NH1/NH2	No	40 OD1/OD2-NE/NH1/NH2	24 OD1/OD2-NE/NH1/NH2
