# Supplementary material for: pH-dependent structural dynamics of neuropeptide Y in aqueous solution
Source: PLoS One. 2026 Mar 12;21(3):e0343614. doi: 10.1371/journal.pone.0343614 (PMC12981483; doi:10.1371/journal.pone.0343614)
Supplement: S5 Table — (PDF) [file pone.0343614.s008.pdf]

pH-dependent structural dynamics of neuropeptide Y in aqueous solution

Hoa Thi Nguyen,[1,2] *Marc Spehr,*[2,3] *Ana-Nicoleta Bondar,*[1,4*] *Paolo Carloni*[1,2,5*]

[1]Forschungszentrum Jülich, Computational Biomedicine, INM-9, Wilhelm-Johnen Straße, 52428 Jülich, Germany

[2]Research Training Group 2416 MultiSenses – MultiScales, RWTH Aachen University, 52074 Aachen, Germany

[3]RWTH Aachen University, Institute for Biology II, Department of Chemosensation, Worringerweg 3, D-52074 Aachen, Germany

[4]University of Bucharest, Faculty of Physics, Atomistilor 405, Magurele, Romania

[5]RWTH Aachen University, Molecular Science and Engineering, Aachen, Germany

*Correspondent authors

Supporting Information

Supporting Information Tables

S5 Table. Helical secondary structure content in the last 110 of standard MD simulations (R#1-3) and for the last 40 ns (R#1and R#2) and 30 ns (R#3) of the constant pHD simulations.

Type of simulations		Residues of the α-helical segment	
		Always within the α-helix	Transient extensions<br>(that is, at times during the dynamics)
Standard MD			
R#1		Glu15-Ile31	Thr32-Arg35
R#2		Ala14-Gln34	none
R#3		Ala14-Arg33	Gln34-Arg35
Constant pH MD			
pH = 7	R#1	Glu15-Ile31	Thr32
	R#2	Glu15-Ile31	Thr32
	R#3	Glu15-Thr32	none
pH = 6	R#1	Glu15-Thr32	Arg33-Arg35
	R#2	Glu15-Ile31	Thr32
	R#3	Glu15-Thr32	Arg33-Gln34
pH = 5	R#1	Glu15-Arg33	Gln34
	R#2	Glu15-Ile31	Thr32
	R#3	Glu15-Ile31	Thr32
pH = 4	R#1	Glu15-Leu30	Ile31
	R#2	Glu15-Ile31	None
	R#3	Glu15-Gln34	Arg35
pH = 3	R#1	Glu15-Thr32	Arg33-Gln34
	R#2	Glu15-Ile31	Thr32-Arg33
	R#3	Glu15-Thr32	Arg33-Arg35
