# Supplementary material for: pH-dependent structural dynamics of neuropeptide Y in aqueous solution
Source: PLoS One. 2026 Mar 12;21(3):e0343614. doi: 10.1371/journal.pone.0343614 (PMC12981483; doi:10.1371/journal.pone.0343614)
Supplement: S1 Text — (PDF) [file pone.0343614.s009.pdf]

pH-dependent structural dynamics of neuropeptide Y in aqueous solution

Hoa Thi Nguyen,[1,2] Marc Spehr,[2,3] Ana-Nicoleta Bondar,[1,4] Paolo Carloni[1,2,5*]*

[1]Forschungszentrum Jülich, Computational Biomedicine, INM-9, Wilhelm-Johnen Straße, 52428 Jülich, Germany

[2]Research Training Group 2416 MultiSenses – MultiScales, RWTH Aachen University, 52074 Aachen, Germany

[3]RWTH Aachen University, Institute for Biology II, Department of Chemosensation, Worringerweg 3, D-52074 Aachen, Germany

[4]University of Bucharest, Faculty of Physics, Atomistilor 405, Magurele, Romania

[5]RWTH Aachen University, Molecular Science and Engineering, Aachen, Germany

*Correspondent authors

Supporting Information

Supporting Information Text

S1 text. H-bond networks

We describe here the intramolecular H-bond network using as criterion for the H-bond angle 20^0 or less (Figs 5, 6B, 7B (main text); S2-S4 Tables). The residues can form direct H-bonds or mediated by 1, 2, 3 water molecules.

Asp6 side chain H-bonds to **Ser3** side chain across all pH values but pH 3. These H-bonds are direct at pH 7 to 5. **Glu10** side chain H-bonds to (i) **Arg25**. The H-bonds are direct at some pH values; (ii) **Asp11** side chain at pH 7 and 6. These are water-mediated H-bonds. **Asp11** side chain H-bonds also to **Arg25** side chain at pH 7. **Glu15** side chain H-bonds to **Arg19** side chain at all pH values except 3. **Asp16** side chain forms direct H-bonds with **Arg19** side chain at all pH values.

The plot indicates a propensity to form intramolecular hydrogen bonds during deprotonation, a trend already observed using the other criterion for the H-bonds. It also reveals relatively few direct hydrogen bonds between the side chains of the peptide. Thus, as it might be expected, polar and charged side chains tend to interact with water.
